# Optofluidic Single-Cell Genome Amplification of Sub-micron Bacteria in the Ocean Subsurface

**DOI:** 10.3389/fmicb.2018.01152

**Published:** 2018-06-08

**Authors:** Zachary C. Landry, Kevin Vergin, Christopher Mannenbach, Stephen Block, Qiao Yang, Paul Blainey, Craig Carlson, Stephen Giovannoni

**Affiliations:** ^1^Department of Microbiology, Oregon State University, Corvallis, OR, United States; ^2^Institut für Umweltingenieurwissenschaften, ETH Zurich, Zurich, Switzerland; ^3^East China Sea Fisheries Institute, Chinese Academy of Fishery Sciences, Shanghai, China; ^4^Department of Biological Engineering, Broad Institute of MIT and Harvard, Cambridge, MA, United States; ^5^Department of Ecology, Evolution and Marine Biology, University of California, Santa Barbara, Santa Barbara, CA, United States

**Keywords:** single-cell genomics, E01-9C-26, marine microbiology, microfluidics, optofluidics, bermuda-atlantic time-series

## Abstract

Optofluidic single-cell genome amplification was used to obtain genome sequences from sub-micron cells collected from the euphotic and mesopelagic zones of the northwestern Sargasso Sea. Plankton cells were visually selected and manually sorted with an optical trap, yielding 20 partial genome sequences representing seven bacterial phyla. Two organisms, E01-9C-26 (*Gammaproteobacteria*), represented by four single cell genomes, and Opi.OSU.00C, an uncharacterized *Verrucomicrobia*, were the first of their types retrieved by single cell genome sequencing and were studied in detail. Metagenomic data showed that E01-9C-26 is found throughout the dark ocean, while Opi.OSU.00C was observed to bloom transiently in the nutrient-depleted euphotic zone of the late spring and early summer. The E01-9C-26 genomes had an estimated size of 4.76–5.05 Mbps, and contained “O” and “W”-type monooxygenase genes related to methane and ammonium monooxygenases that were previously reported from ocean metagenomes. Metabolic reconstruction indicated E01-9C-26 are likely versatile methylotrophs capable of scavenging C1 compounds, methylated compounds, reduced sulfur compounds, and a wide range of amines, including D-amino acids. The genome sequences identified E01-9C-26 as a source of “O” and “W”-type monooxygenase genes related to methane and ammonium monooxygenases that were previously reported from ocean metagenomes, but are of unknown function. In contrast, Opi.OSU.00C genomes encode genes for catabolizing carbohydrate compounds normally associated with eukaryotic phytoplankton. This exploration of optofluidics showed that it was effective for retrieving diverse single-cell bacterioplankton genomes and has potential advantages in microbiology applications that require working with small sample volumes or targeting cells by their morphology.

## Introduction

Single-cell genomics (SCG) and metagenomics are well-proven and effective sources of insight into microbial processes in nature. Single cell genome amplification by optofluidics is a relatively underutilized technology that uses manual sorting with an optical trap to retrieve genomes from low volume cell suspensions. Cells or small cell aggregates that have been injected into a microfluidic chip are selected by the operator on the basis of morphology or other characteristics visible through a microscope, and then moved to reaction chambers within the chip, where the genome amplification is carried out. The small reaction volume (80 nL) reduces both reagent contamination and reagent costs (Marcy et al., 2007; Landry et al., 2013). Aspects of optofluidics that can be an advantage in some applications include very small sample and reaction volumes and visual cell selection (Marshall et al., 2012). Despite potential advantages in some applications, single cell genome amplification by optofluidics has remained relatively rare because fluorescence-activated cell sorting (FACS) approaches using flow cytometry have been very successful and have a much higher throughput rate.

Microfluidics has a long history of success in single-cell analyses, including many examples of single-cell genomics (Blainey et al., 2011, p. 9; Youssef et al., 2011; Marshall et al., 2012; Pamp et al., 2012; Dodsworth et al., 2013, p. 9; Yu et al., 2014). A number of studies have leveraged optofluidics to produce single-cell genomes, often selecting cells with identifiable morphologies. In one early example, the extremely small cells of a novel ammonia-oxidizing *Thaumarchaeota* were selected from an enrichment culture through the use of optofluidics. Examination of the resulting genome assembly was able to demonstrate that these cells contained a number of characteristics specific to their low salinity niche, setting them apart from any previously sequenced members of the Marine Group 1 *Thaumarchaeota* (Blainey et al., 2011). In a subsequent study, entire clonal filaments of intestinal symbionts were sorted on the basis of their filamentous morphology, allowing nearly-complete genome assemblies to be produced. These genomes were able to shed light on the molecular machinery of these cells, providing a mechanistic basis for some of the first plausible explanations of cell differentiation strategies and host-specific interactions in this group. An optofluidic approach also allowed a group of students to amplify the first genomes from sulfide-oxidizing *Thiovulum* cells, selected on the basis of their large cell size and distinctive shape. This provided the first genomic insight into a widely-distributed group of bacteria that had previously evaded any attempt at axenic cultivation (Marshall et al., 2012).

**Figure 1:**
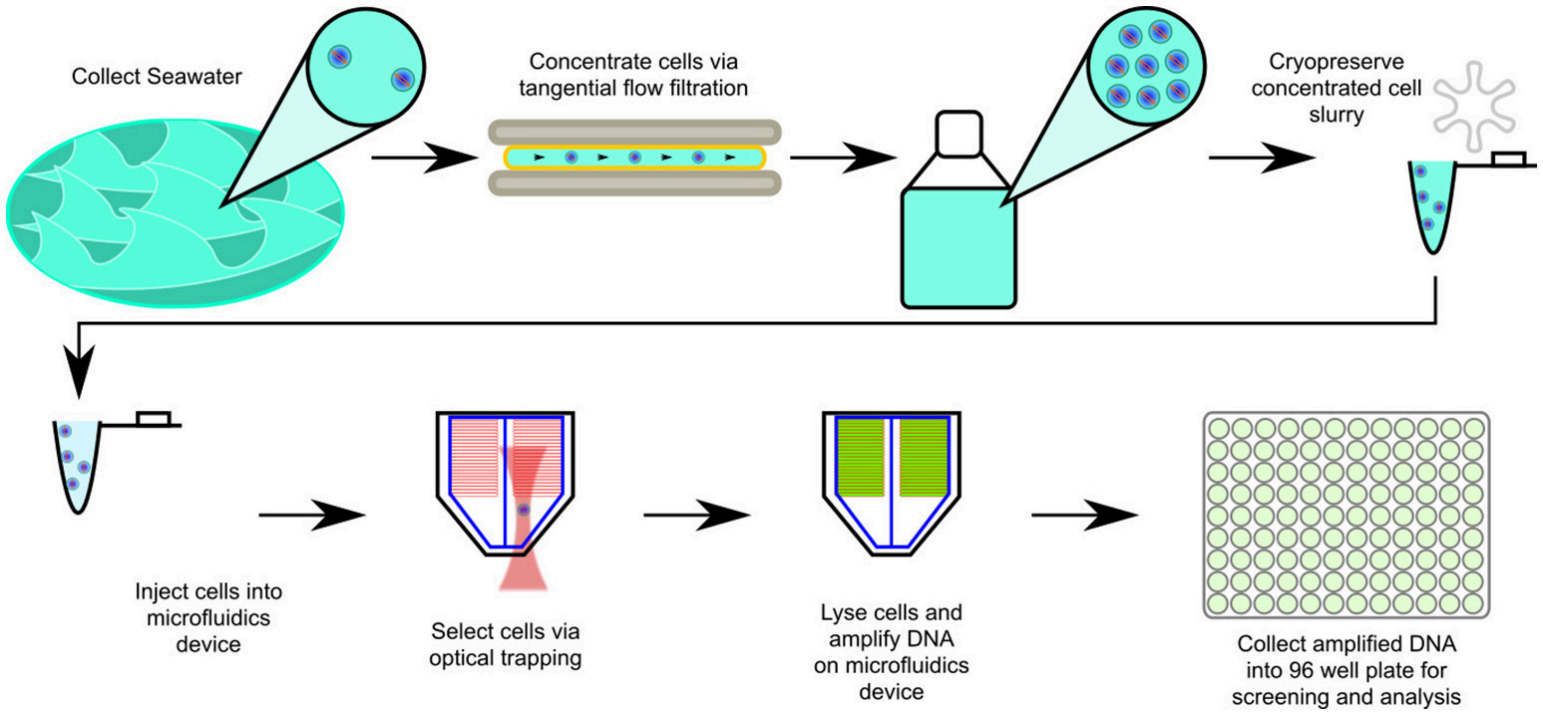
Scheme for optofluidic single cell genome amplification from concentrated marine bacterioplankton samples. In the cell sorts reported here, the operator visually targeted submicron cells. For a more detailed explanation of the technology, please consult (Landry et al., 2013).

In this study we explored the application of optofluidics to marine plankton, testing refinements to our optical trapping design and optimized multiple-displacement amplification (MDA) reaction conditions (Landry et al., 2013). We targeted sub-micron bacterioplankton cells in samples collected from 20 to 250 m during the period of summer stratification in the northwestern Sargasso Sea. A description of our workflow can be seen in Figure 1. Twenty MDA reaction products from seven bacterial phyla were selected for sequencing. Several of the genomes were the first of their types to be sequenced and were examined in detail. Published environmental amplicon diversity data was used to establish the significance of these cell types in ocean ecosystems. Metabolic reconstruction identified features of these cells that suggest specialization. The findings show that optofluidics was effective in this application, and may have future uses in microbiology when small sample size and cell visualization are important factors.

## Materials and methods

### Seawater samples

For this study we used sample volumes of 20–50 μl, at cell concentrations of 10^6^–10^7^ cells/ml. These subsamples were obtained from high-volume tangential flow filtration collections that were being collected for other research purposes and provided a convenient source of ocean gyre plankton cells for optofluidics. Cell suspensions were collected from Hydrostation S (32° 10′N; 64° 30′W) in the western Sargasso Sea on two separate cruise dates (03/10/2011, 07/02/2015) and used on subsequent cell sorts in 2013, 2014 and 2015. Concentrated cell solutions for single-cell genomics were produced by Millipore Pellicon tangential flow filtration (TFF). Prior to filtration, TFF cartridges were washed according to the manufacturer's instructions using solutions of 0.1N NaOH and 0.1N H_3_PO_4_, and flushed with 10 L of deionized water. For the 03/10/2011 samples, 460 L of water was taken from 250 m depth on two consecutive casts, and concentrated to ~200 ml using a Millipore Pellicon 2 TFF cassette cartridge with a 30 kDa size cutoff (regenerated ultracellulose, 0.5 m^2^ surface area). 10 ml aliquots of concentrate were flash frozen in liquid nitrogen, using 10% 0.1 μM filtered DMSO as a cryopreservative. Aliquots were stored at −86°C until use. For the 07/02/2015 samples, 230 L of water was taken from a single cast to 20 m depth, and concentrated to 500 ml using a Millipore Pellicon 2 Maxi cassette cartridge with a 30 kDa size cutoff (regenerated ultracellulose, 2.5 m^2^ surface area). 1 ml aliquots were made using 10% GlyTE buffer (20 ml 100x TE Buffer pH 8.0, 60 ml deionized water, 100 ml molecular grade glycerol; final solution filtered through 0.2 μm syringe filter; Stepanauskas, 2013). Aliquots were frozen in a −86°C freezer using a Mr. Frosty cryopreservation bath, previously stored at −86°C. Frozen aliquots were stored at −86°C until use.

### Single-cell isolation

A 1:1 mixture of the aforementioned cell suspension with a sorting buffer was prepared before sorting. For samples sorted in 2013, prior to sorting, 1 ml of 1x phosphate-buffered saline was amended with 8 μl of 10% Pluronic F-127 and 5 μl of 20 mg/ml molecular-grade bovine serum albumin (BSA). For samples sorted in 2014 and 2015, a zwitterionic buffer was used to help alleviate “sticking” or surface interactions of cells with the inside of the microfluidics device. The initial osmolyte buffer consisted of: 50 mM TAPS buffer, and 500 mM glycine betaine adjusted to pH 8.0 using sodium hydroxide. Prior to sorting, 1 ml of the buffer was amended with 8 μl of Pluronic F-127 and 10 μl 20 mg/ml BSA. Buffer solutions were filtered using a 0.2 μm syringe filter and UV-sterilized for 45 min prior to use. 20–50 μl of the 1:1 buffered cell suspension was used at a time. The buffered cell suspension was injected to a pb_48x_v4 microfluidics device manufactured by Stanford University Microfluidics Foundry using a 0.17” id piece of Tygon tubing fitted with a blunt-end syringe needle and a 0.20” id hollow stainless steel pin. Prior to cell injection, the microfluidics device was UV-treated for 45 min and sample lines of the device were flooded with chip diluent buffer.

Optical isolation was performed using a Leica DMI6000B inverted microscope equipped with combination phase-contrast/fluorescence optics, giving preference to sub-micron cells. Optical isolation was performed using a 976 nm wavelength infrared laser fitted with a customized pinhole spatial filter producing a circular beam with a Gaussian energy profile and a diameter of ~8 mm. Beam steering was performed using 3 dielectric broadband mirrors selected for their high reflectivity at the laser wavelength, and a corresponding periscope assembly and kinematic mount. An achromatic lens with a focal length of 150 mm was mounted to the mirror house at the beam entrance using an adjustable threaded tube mount. The purpose of these optics was to correct the beam diameter and collimation of the laser as it passed through the internal fluorescence optics of the Leica DMI6000B, replacing corrective optics previously installed at the factory. For more details concerning the optical setup, please refer to Landry et al. (2013).

### Whole-genome amplification

Lysis, neutralization, and whole-genome amplification (WGA) reactions were performed within the pb_v4_48x microfluidics device. MDA using Phi29 polymerase was used for all WGA reactions. Lysis and neutralization chambers within the device each had a volume of 3.5 nL and the reaction chamber had a volume of 60 nL. Four separate protocols were used to execute the whole-genome amplification reactions. Complete reaction details for all sets of reactions are found in the Data Sheet 1. For all reaction conditions, lysis solutions were injected and allowed to stand at room temperature for 10 min, after which they were neutralized using an appropriate stop solution. Following neutralization, whole-genome amplification reagents were added and the reaction was allowed to proceed for 16–20 h at 30°C. Reaction solutions and conditions specific to each relevant set of dates are described in Data Sheet 2. Following reaction completion, WGA product for each individual chamber was recovered into a final volume of 15 μl of Tris/EDTA/Tween-20 buffer. All solutions were filtered through a 0.2 μm filter prior to use, and all solutions were UV-treated for 45 min on ice unless otherwise noted. For additional information concerning the function of the microfluidics device please see Landry et al. (2013).

### Quantitative PCR and screening of WGA reaction products

Reaction products were screened by quantitative polymerase chain reaction (QPCR), using universal 16S ribosomal RNA (rRNA) primers to get a measure of 16S copy number as a proxy for reaction success. Three separate protocols were used for QPCR. For all QPCR protocols, ABI PowerSYBR mix was used according to the instructions. For all screenings 20 μl reaction volumes were used (2 μl template into 18 μl of reaction master mix) with a single PCR reaction for each WGA reaction product. All screenings were performed in 96-well plates, with fluorescence readings taken at the end of the extension step. For all assays, a PGEM T-EZ vector with a ligated *Escherichia coli* 16S rRNA gene was used as a standard. Conditions specific to each screening protocol are detailed in the Data Sheet 1. Standards and reactions with low melting temperatures (as determined by disassociation curves) were excluded from the analysis. All standard curves had R^2^ of 0.95 or higher. Reactions passing the detection threshold were cleaned using a Qiagen QiaQuick PCR Clean-up kit according the manufacturer's instructions and sent for Sanger sequencing in Oregon State University Center for Genomic Research and Biocomputing (CGRB) core facilities. Primer sequences were removed from either end for each sequence, and the longest contiguous stretch of bases having a Phred quality score higher than 10 and a mean quality score of at least 20 was kept for further analyses. Sequences with < 100 bp of high-quality sequence were discarded. 16S sequences for 19 SAGs have been deposited in GenBank under accession numbers MG736601-MG736619. The 16S sequence from SAG Met.OSU.00A was excluded from the GenBank submission due to length requirements.

### Phylogenetic screening and 16S amplicon abundance

**Figure 2:**
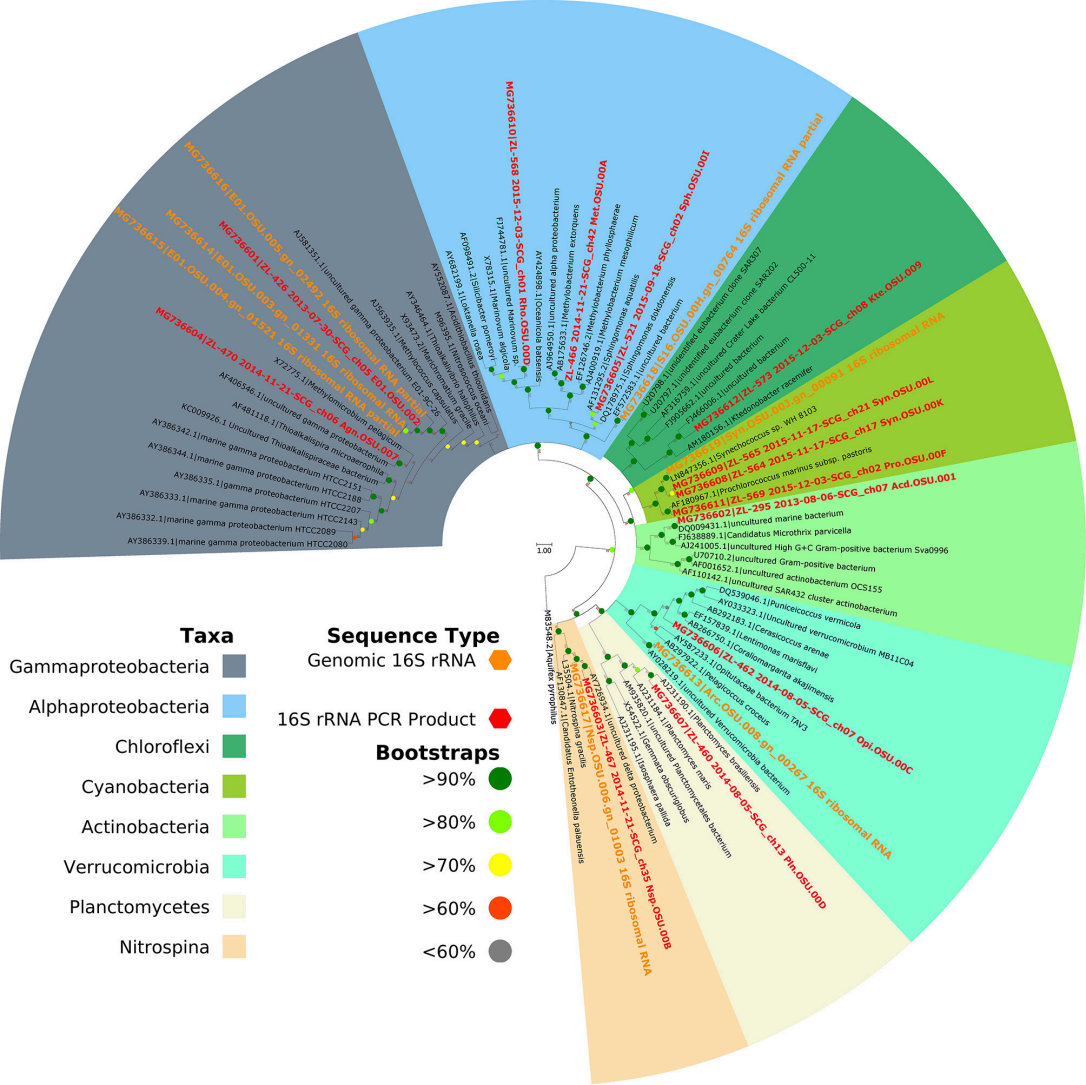
Phylogeny of SAG 16S rRNAs. Clades are color-coded by taxonomy as marked in the key. The scale bar represents 0.03 substitutions per site. Cells from a total of 7 phyla are represented in our data set, indicating that optofluidics is compatible with a number of cell types.

Prior to genome sequencing, the phylogenetic affiliation of each individual WGA product was assessed by comparing PCR-amplified 16S rRNA sequences to reference databases. For preliminary classification, each sequence was initially compared to the most current version of the Silva non-redundant 16S SSU ribosomal RNA sequence, using the Silva web aligner with the default settings and the “search and classify” option (Quast et al., 2013). The SAG sequences, together with the reference sequences, were aligned with the SINA aligner using a PT-Server built from the SSURef_NR99_128_SILVA database. Columns containing nothing but gaps were removed using TrimAl (option: -noallgaps; Capella-Gutiérrez et al., 2009) and the alignment was assessed using Zorro (Wu et al., 2012), masking columns with a weight of < 0.5. The masked alignment was used with IQTree to produce the raw tree file (options: -m TESTNEW -alrt 1,000 -bb 1,000; Nguyen et al., 2015; Hoang et al., 2017; Kalyaanamoorthy et al., 2017). ETE3 (Huerta-Cepas et al., 2016a) was used to create the publication tree seen in Figure 2. The phylogenetics software suite ARB (Ludwig et al., 2004) was used to compare the 16S PCR sequence to a version of the Silva 100 non-redundant 16S SSU ribosomal RNA sequence database containing a published reference tree of marine bacterioplankton that has been used in previous deep amplicon sequencing surveys of the Sargasso Sea (Giovannoni and Vergin, 2012; Vergin et al., 2013, 2017). Alignment was performed using the ARB structural aligner in conjunction with a PT-Server built against the SSURef_NR99_100_SILVA taxonomy, which served as the base for the Vergin et al. (2017) reference tree. Amplicon abundance plots were produced using the Ocean Data View software (Schlitzer, 2002) in conjunction with count data from the environmental 16S amplicon dataset of Vergin et al. (2017). For additional methodology concerning the analysis of this amplicon dataset, please consult Vergin et al. (2017). Counts of 16S genes for the E01-9C-26 clade from the publicly-available TARA Oceans dataset were also used to confirm the abundance of this group in the deep ocean (Sunagawa et al., 2015).

### Phylogenomic placement of E01-9C-26 SAGs

Amino acid sequences from 31 *Gammaproteobacterial* genomes were collected from the NCBI databases for phylogenomic analysis. All four of the E01-9C-26 SAGs were also included. *Alteromonas napthalenivorans* was used as the outgroup. Phylogenomic trees and orthology were determined with a modern reimplementation of the Hal pipeline workflow (Robbertse et al., 2011; Grote et al., 2012; Brown et al., 2016; Landry et al., 2017). A maximum likelihood phylogeny was constructed based on 179 single-copy proteins and 40,074 amino acid residues, allowing for 20% missing data.

### Whole-genome sequencing

Following QPCR screening and quality control, 20 SAGs were selected via their taxonomic sequence affiliation with phylogenetic nodes of interest from the dataset of Vergin 2017 (Giovannoni and Vergin, 2012; Vergin et al., 2013, 2017). A detailed breakdown of taxonomic affiliation, statistics, and assembly information is available in Table 1. Phylogenetic placement of all SAGs can be seen in Figure 2. All 20 samples were sequenced with Illumina MiSeq technology. Indexed libraries for all products were prepared directly from primary amplification products using the NexteraXT protocol by the Oregon State University Center for Genome Research and Biocomputing (CGRB) core facility, with the exception of Acd.OSU.001. Two libraries were prepared for AcdOSU.001, one using the NexteraXT protocol, and a second using TruSeq reagents in conjunction with the pooled product of 3 secondary MDA reaction products produced using the Qiagen Repli-G Single-cell kit in 40 μl reactions. The indexed libraries for assemblies Acd.OSU.001 (both libraries), E01.OSU.002, E01.OSU.003 E01.OSU.004, E01.OSU.005, and Nsp.OSU.006 were sequenced on a single lane of MiSeq paired-end 300 bp reads. The indexed libraries for assemblies Agn.OSU.007, Arc.OSU.008, Kte.OSU.009, Met.OSU.00A, Nsp.OSU.00B, Opi.OSU.00C, Pln.OSU.00D, Rho.OSU.00G, S16.OSU.00H, Pro.OSU.00I, Syn.OSU.00J, Syn.OSU.00K, and Syn.OSU.00L were pooled and sequenced together on a single lane of paired-end MiSeq 250 bp reads. Adapters and library indices were trimmed by the CGRB bioinformatics group.

**Table 1:** Assembly statistics and information for each SAG in this study.

**SAG Name**	**Taxonomy (Silva)**	**Depth (m)**	**N50**	**Assembly size**	**Fractional Completeness (CheckM)**	**Fractional Completeness (CheckM)**	**Fractional Strain heterogenenity (CheckM)**	**Estimated Genome Size (CheckM)**
Opi.OSU.00C	Opitutae	250	16,460	2,077,522	0.78	0.00	0.00	2.65E+006
E01.OSU.003	E01-9C-26 marine group	250	4,189	3,328,816	0.66	0.06	0.13	5.05E+006
E01.OSU.005	E01-9C-26 marine group	250	5,014	2,696,361	0.54	0.03	0.20	4.98E+006
Acd.OSU.001	Sva0996 marine group	250	2,517	1,422,628	0.48	0.03	0.00	2.98E+006
E01.OSU.004	E01-9C-26 marine group	250	3,896	1,851,821	0.39	0.00	0.00	4.76E+006
Nsp.OSU.006	Nitrospina	250	1,981	1,282,535	0.27	0.00	0.00	4.72E+006
S16.OSU.00H	SAR116 clade	20	3,512	789,214	0.27	0.00	0.00	2.96E+006
Arc.OSU.008	Arctic97B-4 marine group	250	5,470	1,518,022	0.13	0.00	0.00	1.17E+007
Agn.OSU.007	AEGEAN_245	250	7,532	246,227	0.10	0.00	0.00	2.38E+006
Pro.OSU.00F	Prochlorococcus	20	631	72,564	0.06	0.00	0.00	1.29E+006
Nsp.OSU.00B	Nitrospina	250	5,581	639,460	0.05	0.00	0.00	1.24E+007
Pln.OSU.00D	Planctomyces	250	1,643	797,959	0.04	0.00	0.00	1.91E+007
Syn.OSU.00J	Synechococcus	20	786	164,756	0.04	0.00	0.00	4.69E+006
E01.OSU.002	E01-9C-26 marine group	250	549	440,564	0.02	0.00	0.00	2.34E+007
Kte.OSU.009	Ktedonobacterales	20	461	6,202	0	0	0	-
Met.OSU.00A	Methylobacterium	250	7,531	289,220	0	0	0	-
Rho.OSU.00G	Rhodobacteraceae, uncultured	20	850	6,009	0	0	0	-
Sph.OSU.00I	Sphingomonas	20	745	12,452	0	0	0	-
Syn.OSU.00K	Synechococcus	20	828	100,639	0	0	0	-
Syn.OSU.00L	Synechococcus	20	1,393	39,437	0	0	0	-

*Species or group level placements within the Silva taxonomy, and a variety of assembly statistics including estimated SAG completion can be seen here*.

### Assembly, quality control and annotation

For all single-amplified genomes (SAGs), standardized protocols for assembly, quality control, and annotation were followed. Reads from each assembly were initially subjected to quality control and standardized 3-pass digital normalization as described in the ‘Kalamazoo metagenomic assembly protocol' via the khmer package (v2.0; Brown et al., 2013). Prior to digital normalization, reads were interleaved using the “interleave-reads.py” tool included in the khmer package and were subjected to quality control via the FastX package (v0.0.13.2; “fastq_quality_filter,” options: -Q33 -q 30 -p 50). Paired and single-ended reads were then extracted using the “extract-paired-reads.py” tool included in the khmer package. The paired-end reads were then subjected to a first round of digital normalization to 20x coverage using the “normalize-by-median.py” tool (options: -k 20 -C 20 -N 4 -x 5e8 -p –savehash), as were the single-ended reads (options: -C 20 –savehash normC20k20.kh –loadhash). Low abundance kmers were removed using the “filter-abund.py” tool included in the khmer package and paired-end reads were extracted. The remaining sets of paired-end (options: -C 5 -k 20 -N 4 -x 5e8 –savehash) and single-ended reads (options: -C 5 –savehash normC5k20.kh –loadhash) were then normalized again to 5x coverage. Following digital normalization, reads were assembled using the SPAdes assembler (v3.6.2; with the options –sc and -k “21,33,55,77,99,127”) with both the paired-end and single reads reserved after digital normalization as input. Contigs were re-named with short names derived from their assembly names and renamed contigs were automatically annotated using the PROKKA software package for prokaryotic annotation (Seemann, 2014; v1.11). PROKKA was run with default options, specifying the likely envelope structure of the organism based on assessed taxonomy (gram + or -). Quality control to detect possible contaminant sequences was performed as described in the c*ontaminant detection* section below. A genbank-formatted file containing PROKKA-annotated contigs that passed our internal quality control were used in conjunction with the Pathway Tools package to convert EC Numbers and gene descriptor lines into functional annotations corresponding to pathway reactions. The PathoLogic capability of the Pathway Tools (Karp et al., 2015; Caspi et al., 2016) package was used with default options to elucidate potential metabolic pathways. Assemblies for the E01.OSU.005, E01.OSU.003, Opi.OSU.00C, and Acd.OSU.001 SAGs have been deposited into GenBank under the accession numbers PNEU00000000–PNEX00000000.

### Contaminant detection

Principal component analysis (PCA) of tetranucleotide frequencies is a standard data exploration technique for the detection of contaminants in single-cell datasets (Woyke et al., 2009). Principal component analysis was combined with an implementation of the DBSCAN clustering algorithm (Ester et al., 1996) to provide a semi-supervised method of separating outliers or contaminating contigs from the remainder of the assembly. A sweave/knitr-generated (Xie, 2013) document detailing the implementation of this process in the R programming language is included in the supplement, along with a scripted version of the process Data Sheet 3. This combined PCA/clustering approach was used to initially examine our dataset for any potential contaminants. The resulting PCA plots and classifications were then manually inspected to judge confidence in the results. A post-processing step used BLAST results to refine the classification by comparing coding sequences included on spurious contigs against the RefSeq databases and reclassifying these contigs if they matched the taxonomic range of the main cluster.

### Assembly completion and genome size estimates

Following assembly and annotation, genomes were assessed for completeness, contamination and strain heterogeneity via CheckM (Parks et al., 2015). Amino acid sequences from each assembly were assessed using the –lineage_wf option. Additionally, all genes included in validated contigs were subjected to a hmmscan search with default options against the EggNOG v4.5 database (Huerta-Cepas et al., 2016b) of hidden Markov models. Top hits were assigned to each protein. Proteins with top-hitting models falling into the ′original group hierarchies' for any of the clusters of orthologous groups (COGs) included in the Raes et al. (2007) set of ubiquitously-conserved single-copy marker genes were tallied. Genome completion was estimated here as the fraction of this single-copy conserved marker gene set recovered (out of 35 COGs). The inverse of this fraction multiplied by the total length of valid contigs was used to produce a genome size estimate for each assembly. The results of this analysis can be seen in Data Sheet 2.

## Results and discussion

### Whole genome sequencing, assembly, and phylogeny

Optofluidics is a rapidly evolving technology, and therefore in this investigation we explored the operation of a new workstation (described in Landry et al., 2013) and variations in reaction conditions. With the most recent version of the amplification protocol we used (Data sheet 1; Protocol 4), of 89 attempted reactions 26 reactions surpassed the QPCR detection limit of 100 copies/μl, a success rate of 29%. These figures compare well to the success rates of other marine SCG studies using FACS, which report ~ 30% rate of reaction success when using similar amplification and screening protocols (Stepanauskas et al., 2017). The most complete SAG was 78% complete, and all SAGs had estimated contamination of < 10%. The most recent protocols and alternatives that were previously tested can be found in the Data Sheet 1.

Phylogenetic placement revealed the SAGS were from a wide variety of taxa, most of which were identified as abundant plankton types (Table 1). The SAGs spanned 7 phyla, indicating that the lysis and amplification protocols are compatible with a wide variety of cell types. Silva database classifications can be found in Table 1, as well as in Data Sheet 2. Assembly statistics and genome size estimates for each of the 20 genomes are available in Table 1. CheckM gave completion estimates from assembled genomes ranging from below the threshold for completeness detection, to as high as 78% complete. Genome recovery is highly dependent on sequencing depth and varies greatly with reaction chemistry and cell type, but these numbers are within the range of previous reports, with similar reaction chemistries often recovering < 50% of the genomes (Stepanauskas et al., 2017). Nearly all SAGs showed a high degree of fragmentation, which is typical of single-cell assemblies. The mean N50 was 3,578 bp, and the average L50 for all assemblies was 117 contigs.

### E01-9C-26 *Gammaproteobacteria* SAGs

Although data we report indicate E01-9C-26 is abundant throughout the dark ocean, genomes from these cells have not been characterized previously. These organisms were first described in 16S rRNA gene clone libraries from marine sponges, and subsequently were found in marine plankton (Thiel, 2006; Thiel et al., 2007a,b). In a depth profile from the central Mediterranean, E01-9C-26 clones were the only *Gammaproteobacteria* group found at every depth in both DNA and RNA-based clone libraries (Smedile et al., 2015). E01-9C-26 was also reported to be a major contributor to both DNA and RNA libraries from the water column in the Adriatic Sea and was present to a lesser degree in marine snow libraries from the same site (Vojvoda et al., 2014). E01-9C-26 contributed up to 7.3%, and on average, 2–3% of the total bacterial amplicons from the upper mesopelagic at the Bermuda Atlantic Time-series Study (BATS) site (data from Vergin et al., 2017; Figure 4A) and was abundant year-round at that site. Publicly available TARA Oceans amplicon data confirmed that E01-9C-26 is ubiquitously distributed throughout the dark ocean (Data Sheet 4; Sunagawa et al., 2015).

The E01-9C-26 SAGs were estimated to be 54% (E01.OSU.003) and 66% (E01.OSU.005) complete, with estimated sizes around 5 Mbps. 16S rRNA genes from assemblies were confidently placed within the E01-9C-26 *Gammaproteobacterial* group of the Silva 100 taxonomy, related to the cultured species *Thioalkalispira microaerophila* and *Methylococcus capsulatus*. The 16S sequence identities of E01.OSU.003 and E01.OSU.005 with *Thioalkalispira microaerophila* are 90 and 89%, respectively, and 90% to *Methylococcus capsulatus* for both strains.

**Figure 3:**
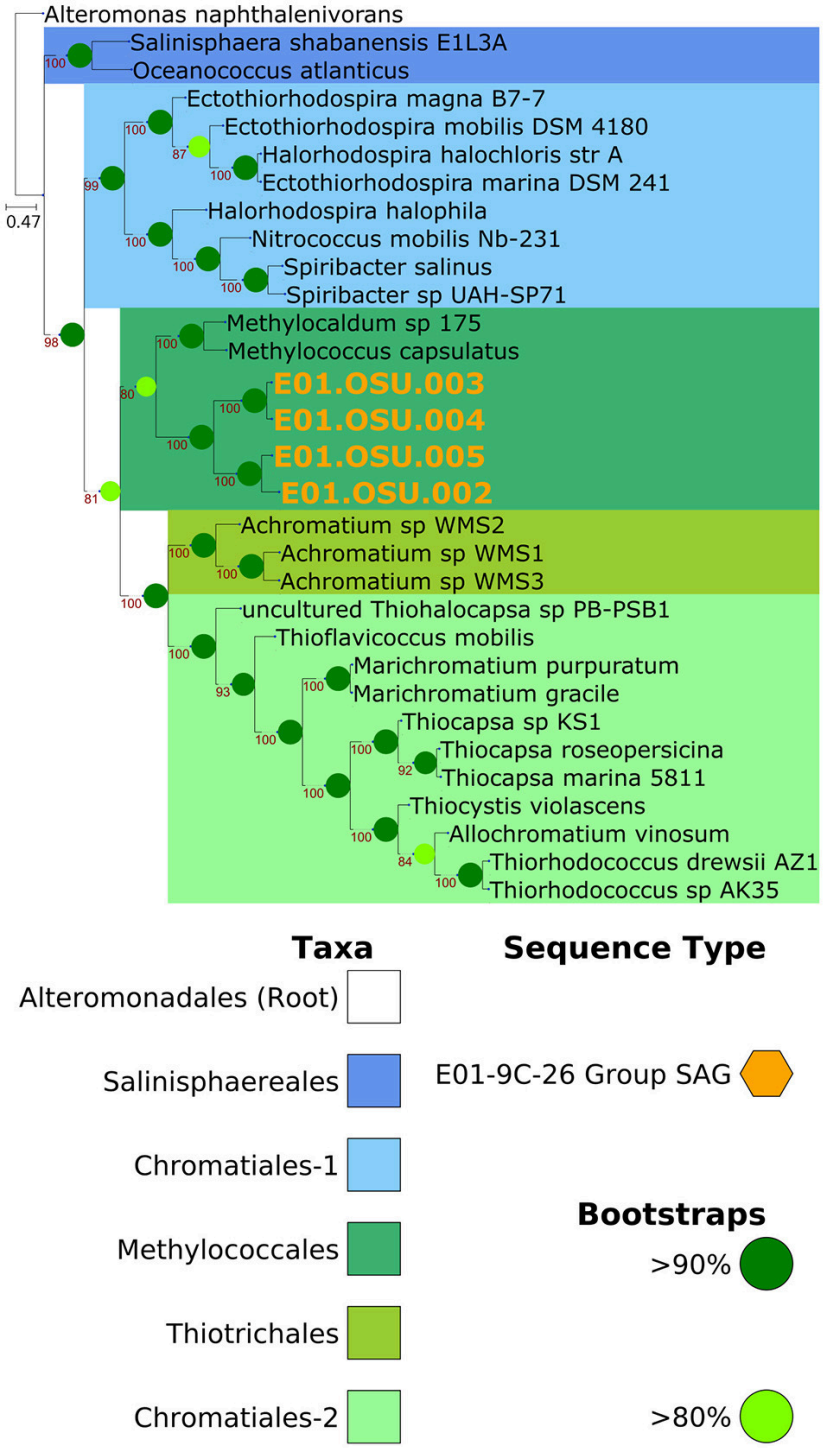
Phylogenomic placement of E01-9C-26 within the Gammaproteobacteria. Placement of the E01-9C-26 clade SAGs indicates it that it is a related group to *Methylococcus capsulatus*. Consistent with this placement, many genes for methylotrophy were observed in the genomes. Tree is based on 142 single-copy conserved protein sequences and 32,829 amino acid residues, with up to 20% missing data allowed.

Phylogenomic analysis confirmed E01-9C-26 as a sister clade to *Methylococcus capsulatus* (Figure 3), which was interesting as metabolic reconstruction indicated these cells are versatile methylotrophs. The genomes encode formaldehyde oxidation proteins, formate dehydrogenase, a PQQ-dependent alcohol dehydrogenase annotated as a methanol dehydrogenase (*mdh*), a complete tetrahydrofolate pathway for methyl-group oxidation, and a several enzymes of the serine cycle, including the key enzyme serine hydroxymethyltransferase.

**Figure 4:**
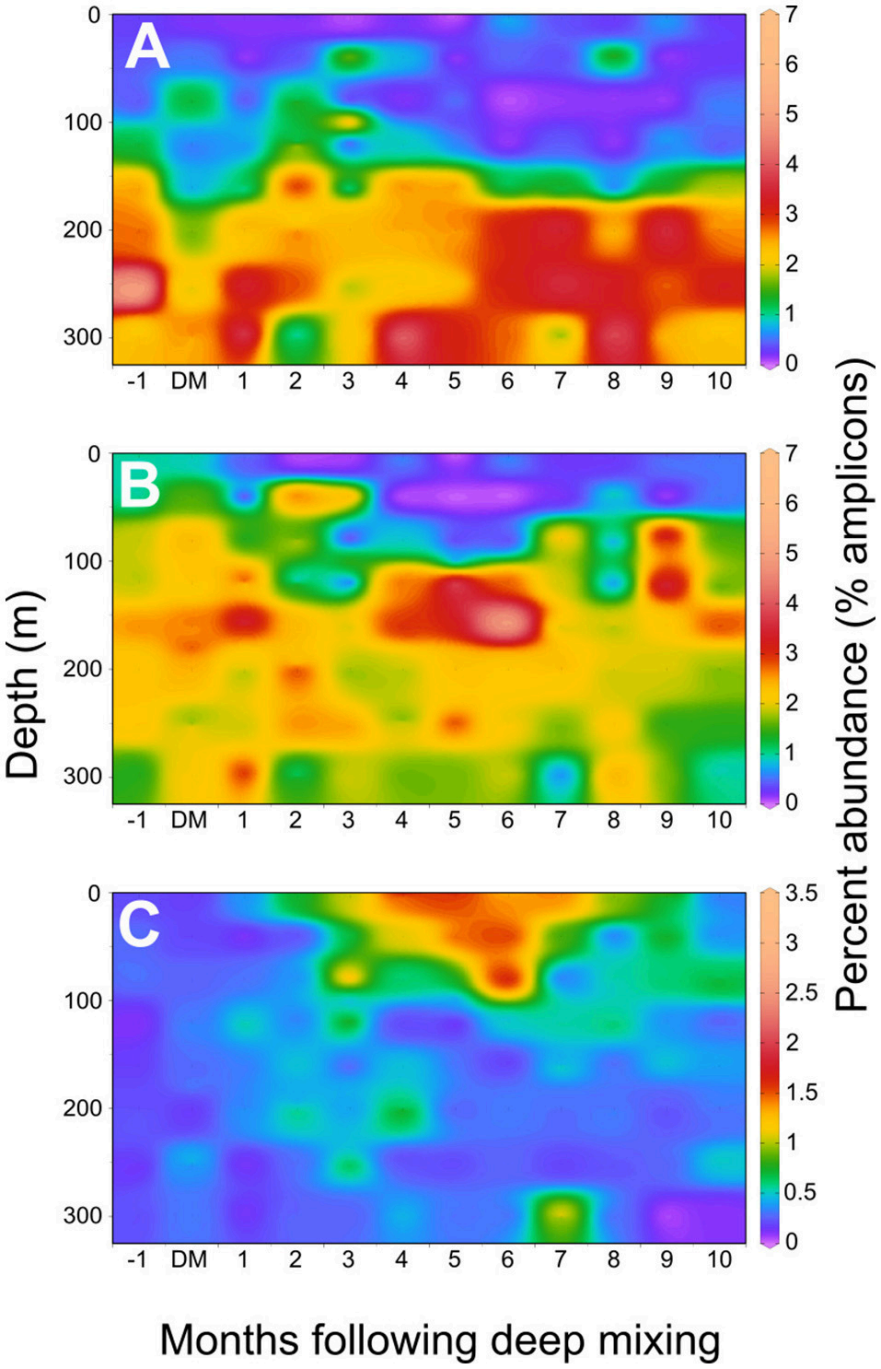
Seasonal distributions of SAG taxa in the western Sargasso Sea. An annual composite of 16S rRNA amplicon data from a BATS 10-year time series were used to plot variation in the upper 300 meters at BATS (data from Vergin 2017; Giovannoni and Vergin, 2012; Vergin et al., 2013). X-axis represents month-relative timing of maximal deep mixing (DM) in each year of the time series record, as previously reported. **(A)** E01-9C-26 marine *Gammaproteobacteria* **(B)**. Sva0996 marine *Acidimicrobiales*, **(C)** *Verrucomicrobia* node 1938, corresponding to the Opi.OSU.00C SAG.

**Figure 5:**
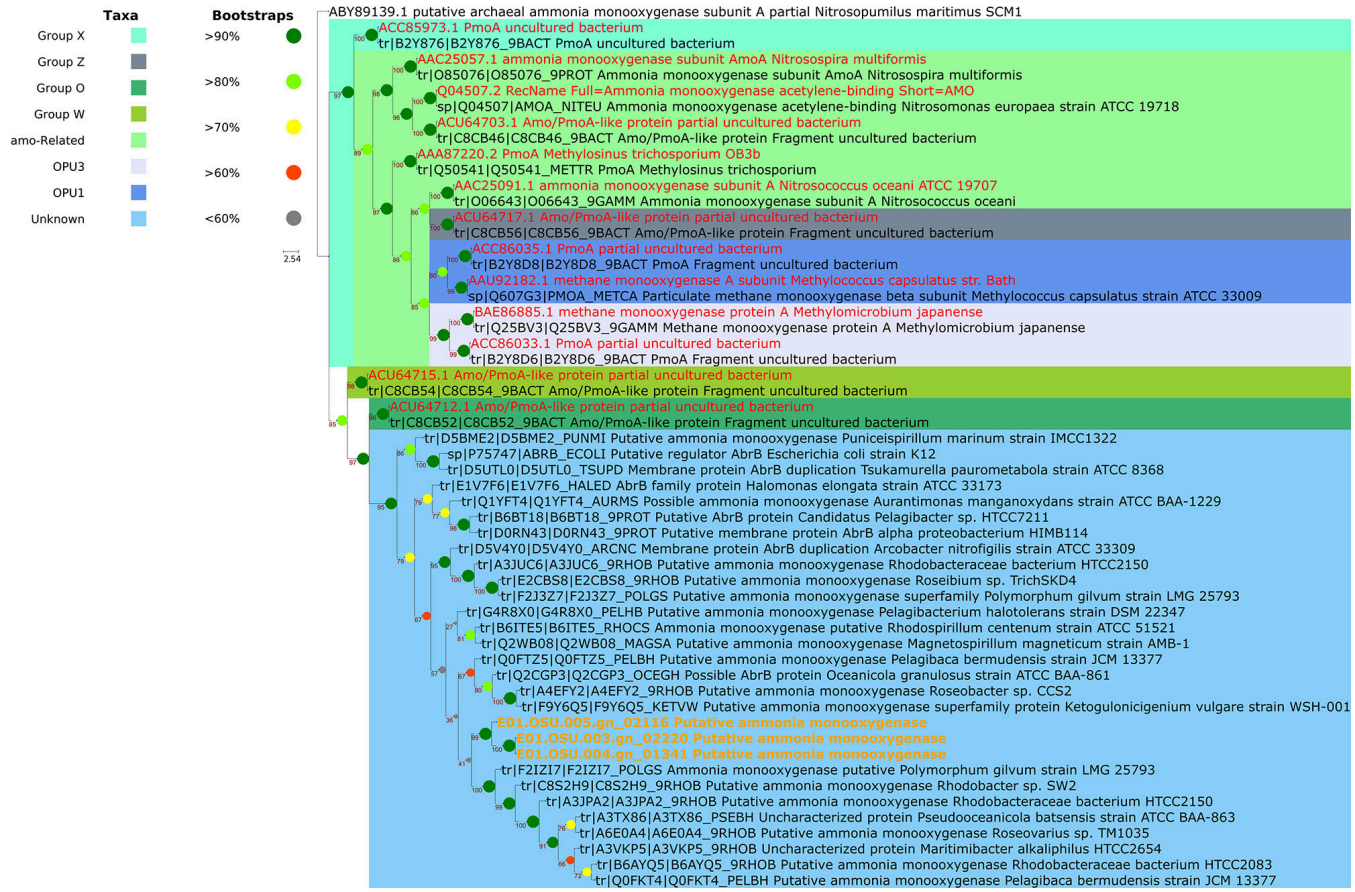
Phylogenetic placement of E01-9C-26 monooxygenase genes annotated as “putative ammonia monooxygenase". The functions of these proteins are unknown, but their relationships to proteins of proven function indicate they may be methane or ammonium monooxygenases. Monooxygenase clades are named according to Tavormina et al. (2010), who reported abundant monooxygenase genes in the mesopelagic of the Sargasso Sea and in California coastal samples. Related sequences are present in a number of cultured oligotrophic marine bacteria in the High Throughput Culturing Collection (HTCC).

Also found in E01-9C-26 genomes were monooxygenases that grouped phylogenetically with the “O” and “W” monooxgenases previously reported by Tavormina et al. (2010) from dark ocean habitats. The most frequent annotations of these genes are methane or ammonium monooxygenase, but it is possible that these could be monooxygenases with other specificities. The phylogeny in Figure 5 shows that similar proteins are found in a variety of cultured marine bacterial strains, where their functions are unknown.

**Figure 6:**
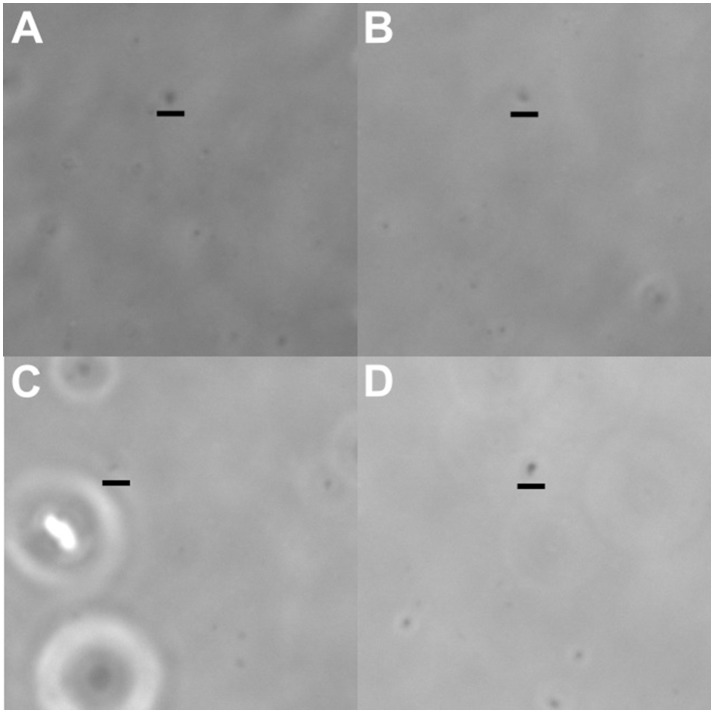
Images of cells selected to produce the whole-genome amplification products discussed in this paper. All cells had very small morphologies, as is typical for pelagic, free-living bacterioplankton. **(A)** E01.OSU.003, **(B)** E01.OSU.005, **(C)** Acd.OSU.001, **(D)** Opi.OSU.00C. Scale bars are 2μm, and have been placed directly below the cells in question.

**Figure 7:**
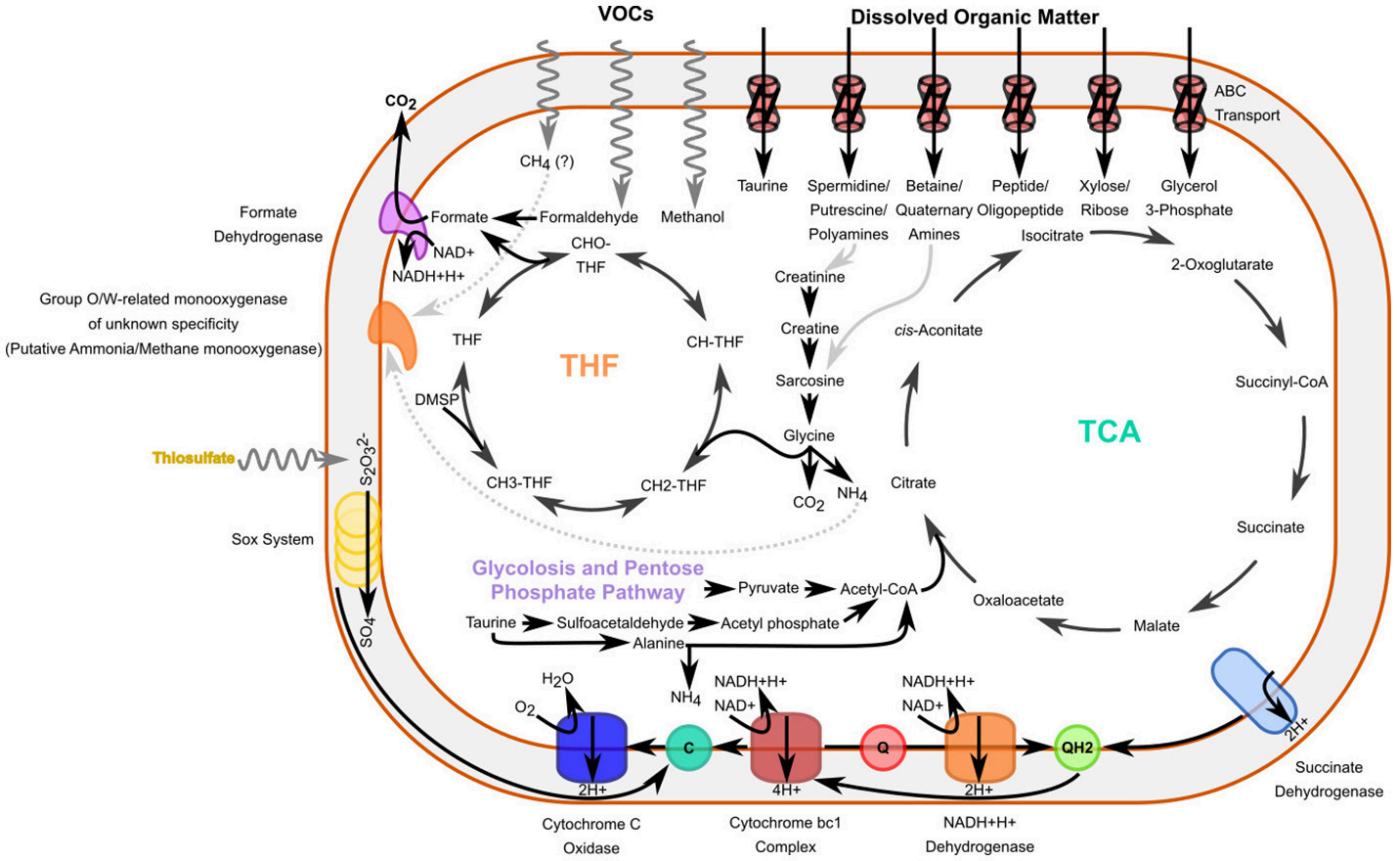
Cell diagram outlining some of the probable metabolic features of a E01-9C-26 Gammaproteobacterial cells. These cells appear to have the ability to degrade or assimilate a methylated compounds, as well as amines, and may additionally have the ability to oxidize reduced sulfur, as well as use organosulfur compounds for energy or growth. THF: Tetrahydrofolate-mediated oxidation/assimilation pathway for methylated compounds. TCA: Tricarboxylic Acid cycle. Gray pathways indicate plausible sources of some specific compounds.

The E01-9C-26 genomes, which are relatively large, predict many functions in addition to methylotrophy, suggesting a complex lifestyle. A cell diagram highlighting features of their predicted metabolism can be seen in Figure 7. The presence of protein coding sequences for NADH:Ubiquinone oxidoreductase, succinate dehydrogenase, cytochrome bc1, and cytochrome c oxidase complexes of the electron transport chain indicate oxygen respiration, but as described below, the genomes also encode dissimilatory sulfate reduction, suggesting anaerobic respiration might be possible in the absence of oxygen. Genes were found for most enzymes of the tricarboxylic acid cycle (TCA), a full beta-oxidation cycle, and the non-oxidative branch of the pentose phosphate pathway. Epimerases specific to the degradation of D-amino acids suggested these cells might play a role in the remineralization of D- amino acids in the deep ocean. The genomes also encode genes for the degradation of a range of other amino acids and polyamines, as well as the amine compounds taurine, carnitine, urea, creatinine and creatine, which are thought to be produced by marine invertebrates (Webb and Johannes, 1967). These compounds can serve as substrates for microbial growth, and recently some have been proposed to be far more abundant than previously thought. Taurine in particular was recently shown to be excreted in massive amounts by marine crustacea at rates in excess of 1 μmol/gram carbon biomass/hour, suggesting that this compound is a potentially abundant carbon, nitrogen, sulfur, and energy source throughout the marine environment (Clifford et al., 2017).

In addition to dissimilatory sulfate reductase, mentioned above, E01-9C-26 encoded many additional genes that suggest a role in the ocean sulfur cycle. Among these were genes implicated in sulfur oxidation, including *SoxA, SoxY*, and *SoxZ* proteins involved in thiosulfate oxidation (but not *SoxB)*. Recent studies have also suggested that organosulfur compounds may be more widely-utilized by marine bacteria that previously thought (Durham et al., 2015). Many genes involved in pathways for the degradation of organosulfur compounds, such as sulfonates or sulfoamines, were present, including mostly complete pathways for taurine, hypotaurine, sulfoacetate, sulfoacetaldehyde, sulfopropanediol, and sulfolactate degradation as well as the individual enzymes: methanesulfonate monooxygenase, alkanesulfonate monooxygenase and dimethylsulfoniopropionate (DMSP) demethylase.

### *Opitutae*-related SAG Opi.OSU.00C

The cell used to generate the Opi.OSU.00C assembly was collected from 250 m, but an analysis of BATS amplicon data indicated a pronounced peak in the abundance of these cells near the surface in the late spring (1.65% relative amplicon abundance; Figure 4C). Given the seasonality and depth distribution of this group (Figure 4C), it seems probable that Opi.OSU.00C may be adapted to late spring bloom conditions in the northwestern Sargasso Sea, where eukaryotic phytoplankton flourish transiently before stratification and macronutrient depletion limits their growth. In these genomes we found many genes that suggest these cells are adapted to catabolize diverse carbohydrates of eukaryotic origin.

Nearly-complete genomes were obtained from some optically trapped cells. The assembly Opi.OSU.00C, a marine *Verrucomicrobia*, was estimated to be 78% complete with a projected genome size of 2.65 Mbps, and represents the first genome of its kind. Although Opi.OSU.00C is distantly related to marine *Verrucomicrobia* strains *Coraliomargarita akajimensis* and *Puniceicoccus vermicola* (Choo et al., 2007; Yoon et al., 2007; Mavromatis et al., 2010), its genome is much smaller than the 3.75 Mbp genome of its closest cultured relative, *Coraliomargarita*, and also has a much lower GC content: 39% compared to 54% (Mavromatis et al., 2010).

The genomes included transporters for galactopyranose, ribopyranose, xylose, and arabinose as well as enzymes for the degradation of the uronic acid compounds galacturonate, fructuronate, rhamnogalactouronan, and the sugars 1,5-anhydrofructose, sucrose, xylose, allose, mannose, galactose (via galactonate), and 3,6-anhydro-L-galactose. Nearly all of these sugar moieties are well represented in the structural carbohydrate fraction of eukaryotic phytoplankton. Uronic acids are a major constituent of structural polymers from brown, red and green algae, including prasinophytes, which dominate the surface in the late winter mixing event in the Sargasso Sea (Haug et al., 1974; Fichtinger-Schepman et al., 1981; Jaseja et al., 1989; Becker et al., 1994, 1998; Treusch et al., 2012). The sugar monomers xylose, mannose, arabinose, and galactose have been shown to be abundant monomers contributing to the cell walls of a number of algal species, including the major marine primary producer *Emiliana huxleyii* (Fichtinger-Schepman et al., 1981). 1,5 anhydrofructose is a starch derivative and a precursor to the antibacterial secondary metabolite microthecin, produced by some red algal species (Broberg et al., 1996). Furthermore, a partial catabolic pathway for the degradation of the sugar 3,6-anhydro-L-galactose, another central component of the cell walls of red algae, was present in the genome (Yun et al., 2015). Finally, this small cell encoded at least 23 different sulfatase genes. Sulfated polysaccharides are common components of the cell walls of a number of red and brown algae (McCandless and Craigie, 1979).

The genome additionally included a limited number of genes for amine degradation, including genes involved in the degradation of D-arginine, L-citrulline, creatinine, putrescine, spermidine as well as a transporter for unspecified polyamines. Genes for the degradation of the organosulfur compounds sulfocatechol, taurine and sulfolactate were also found.

### Sva0096 marine *Acidimicrobiales* SAG Acd.OSU.001

One of the genomes, Acd.OSU.001, is a member of the Sva0996 marine *Acidimicrobiales* and is closely related to the MedAcidi-G1 group of *Acidimicrobiales* described in Mizuno et al. (2015). Sva0996 marine *Acidimicrobiales* were initially observed in benthic sediment samples from the (Ravenschlag et al., 1999) and were subsequently shown to be prominent in the water column of the Arctic Ocean (Bano and Hollibaugh, 2002). They have been reported to be a major contributor to microbial communities forming within mode-water eddies in the Sargasso Sea (Nelson et al., 2014), and amplicon data indicates that these cells represent a large component of the summer deep chlorophyll maximum (DCM) community at BATS, with peak abundances of up to 6.65% of amplicon sequences at 100 m (Vergin et al., 2017; Figure 4). Interestingly, this organism is abundant in metagenomic data originating from sponge microbial communities, but bias in the commonly used 519R primer leads to their almost total exclusion from corresponding 16S libraries (Fan et al., 2012). The use of the 338RPL primer in the work of Vergin et al. (2017) probably allowed detection of this group. Sva0996 is distinctly different from the canonical *Actinomarina* (SAR432, OCS115) group of marine *Actinobacteria* (Rappé et al., 1999; Ghai et al., 2013). While this is the first single-cell genome to be reported from this group, metagenomic assemblies of a number of members of this group have recently become available (MedAcid-G1, MedAcid-G2, MedAcid-G3; Mizuno et al., 2015).

The Acd.OSU.001 SAG had a genome recovery estimate of 48% and an expected genome size of 2.93 Mbps. This estimate is similar to metagenomic assemblies of related *Acidimicrobiales* from the Mediterranean Sea, which had predicted genome sizes ranging from 1.85 to 2.33 Mbps (Mizuno et al., 2015). GC content for our assemblies was about 43% - within the range of the closest related metagenomic assembly, “MedAcidi-G1” (16S rRNA identity of 99%).

The genomes also included complete or mostly-complete pathways for a number of central metabolic pathways including the glycolysis pathway, pentose-phosphate pathway, a TCA cycle with a glyoxalate bypass, as well as a complete beta-oxidation cycle, and propanoyl-CoA degradation pathway. Acd.OSU.001also appeared to have the ability to degrade a limited range of amines, including a number of common amino acids and the organosulfuramine compounds taurine and hypotaurine. The genomes also encoded proteins for oxidation of the C1 compound formaldehyde (via mycothiol and formate dehydrogenase) and the algal osmolyte DMSP. One feature not previously discussed in the literature is the presence of a bifunctional xylanase/deacetylase in these genomes, as well as genes for xylan and xylose degradation and transport indicating the potential to digest xylan, which represents a major structural compound in a number of algae that are prevalent in the world's oceans. The vertical distribution of Sva0996 at BATS indicated they are most abundant in the region of the DCM (Figure 4B), which is consistent with the predicted capacity of these cells to degrade DMSP, xylan and xylose, which, in the ocean, are mainly phytoplankton products.

## Conclusions

This project aimed to further our knowledge of bacterioplankton diversity by harnessing optofluidics to sequence genomes from uncultured cells. We tested an updated workstation design, and variations on reaction chemistry. Over the long duration of the project other technologies - metagenome assembly and single cell genomics by FACS - matured, making genomes from uncultured cells relatively common. Nonetheless, in this study single-cell genome amplification by optofluidics was effective for the acquisition of genomes from small planktonic cells (Figure 6; Videos S1–S4), and yielded 20 genomes from small bacterioplankton cells that spanned seven bacterial phyla. Genome completeness and contamination are documented here, and compare favorably with alternative approaches (Stepanauskas et al., 2017). It seems likely that in the future this method will be applied in situations where its unique advantages—visual cell selection and small sample size—are important factors.

An examination of amplicon data from the BATS site showed that the genomes we retrieved were from cell types that are common in the euphotic zone and upper mesopelagic, where the samples were collected. Two of the microbial groups, E01-9C-26 *Gammaproteobacteria* and Opi.OSU.00C *Verrucomicrobia*, previously had not been studied by genome sequencing, and thus were analyzed in detail. We also analyzed a third genome, from the abundant, but largely unexamined Sva0096 marine *Acidimicrobiales*, a group for which metagenomic assemblies have only recently become available (Mizuno et al., 2015). For these taxa, the genomes provided evidence of metabolism that could be interpreted as adaptive to niches in the ocean water column. Interpreting microbial genomes in this way is challenging because cells often are eclectic combinations of biochemistry that appear to have evolved to harvest diverse resources. Nonetheless, ecological data have made it clear that plankton communities are highly determined by their environments, a process called environmental filtering (Fuhrman et al., 2006; Fuhrman and Steele, 2008; Fuhrman, 2009; Vergin et al., 2017). These data showed clearly that Opi.OSU.00C *Verrucomicrobia* reach a maximum near the surface in the late Spring (Figure 4C), whereas E01-9C-26 is abundant throughout the dark ocean (4A).

Phylogenomic analysis and genome content supported the conclusion that E01-9C-26 is a methylotroph, but its large genome also encoded genes for catabolizing a wide array of organic carbon compounds, including many amine-containing compounds that are often associated with invertebrate metabolism. These genomes also encoded monooxygenases of unknown function that are ubiquitously distributed in the oceans (Tavormina et al., 2010), and genes for sulfate respiration, suggesting it is able to shift to alternative electron acceptors when oxygen is depleted. All of these adaptations have interesting ramifications for understanding dark ocean ecosystems, where methylotrophy has not received much attention, but invertebrates are important food web constituents.

Opi.OSU.00C *Verrucomicrobia* flourish in the northwestern Sargasso Sea euphotic zone late in the cycle of the spring bloom, a period of transition from eukaryotic phytoplankton dominance to stratification, nutrient limitation, and cyanobacterial dominance (Treusch et al., 2012). In these genomes we found many genes that suggest these cells are adapted to catabolize diverse carbohydrates of eukaryotic origin, leading us to propose their niche is linked to a seasonal cycle in DOM composition.

Our results are genome-enabled hypotheses arising from data gathered with an innovative and underutilized technology. These hypotheses remain to be confirmed by experiments with cells or field studies. Optofluidics was effective in this application, which targeted submicron particles, and will probably remain part of the microbiology toolkit, to be applied to specialized problems where cell visualization and working with small sample sizes are important factors in the experimental design.

## Author contributions

ZL built and maintained the SCG facility at Oregon State University, as well as trained and supervised users and operators. He additionally performed all bioinformatic analyses and interpretation of genomic data. KV collected, processed, analyzed, and interpreted amplicon sequencing samples, work which was invaluable to this study. CM provided extraordinarily diligent laboratory and troubleshooting work with the optofluidics system. The participation of SB was influential in the establishment of the single-cell genomics facility at OSU. QY provided useful and pertinent advice and a careful set of hands during reaction optimization. PB was an invaluable technical resource during the initial construction of the single-cell genomics workstation at OSU. CC was invaluable in interpreting the content of single-isolated genomes with regard to coinciding environmental conditions. The oversight of SG was paramount in the budgeting, inspiration, motivation, implementation and execution of this project. All authors contributed to the manuscript preparation and editing.

### Conflict of interest statement

The authors declare that the research was conducted in the absence of any commercial or financial relationships that could be construed as a potential conflict of interest.

## Supplementary material

Data Sheet 1 Supplementary laboratory methods.

Data Sheet 2 Table with a variety of additional statistics and information for each SAG isolated in this study.

Data Sheet 3 DBSCAN implementation for potential contaminant detection in R. This document describes the semi-supervised method to examine single-amplified genome assemblies for possible contaminants. Includes an R-scripted version of protocol.

Data Sheet 4 Relative abundance of E01-9C-26 Gammaproteobacterial 16S genes from the TARA Oceans dataset. Ribosomal rRNA genes from this group are ubiquitously present in mesopelagic samples throughout the dataset, suggesting that the clade is numerically relevant to this environment throughout the world's oceans.

Video S1 Video of cell sorted for E01.OSU.003 SAG. Cells sorted can be identified by Brownian motion at the center of the frame.

Video S2 Video of cell sorted for E01.OSU.005 SAG. Cells sorted can be identified by Brownian motion at the center of the frame.

Video S3 Video of cell sorted for Acd.OSU.001 SAG. Cells sorted can be identified by Brownian motion at the center of the frame.

Video S4 Video of cell sorted for Opi.OSU.00C SAG. Cells sorted can be identified by Brownian motion at the center of the frame.
