# Supplementary material for: Optofluidic Single-Cell Genome Amplification of Sub-micron Bacteria in the Ocean Subsurface
Source: Front Microbiol. 2018 Jun 8;9:1152. doi: 10.3389/fmicb.2018.01152 (PMC6003095; doi:10.3389/fmicb.2018.01152)
Supplement: Data Sheet 1 — Supplementary laboratory methods. [file Data_Sheet_1.DOCX]

Supplementary Material

**Optofluidic single-cell genome amplification of E01-9C-26 and other sub-micron bacteria specialized to niches in the ocean subsurface**

**Zachary Landry, Kevin Vergin, Christopher Mannenbach, Stephen Block, Qiao Yang, Paul Blainey, Craig Carlson and Stephen Giovannoni^*^**

*** Correspondence:** Stephen Giovannoni: Stephen.Giovannoni@oregonstate.edu

# Supplementary Methods

## WGA REACTION CONDITIONS

### **07/30/2013, 08/06/2013 (Protocol 1):**

The sample sorted was a concentrated cell suspension from the 03/10/2011 cruise, taken from 250 m depth. The lysis solution consisted of a 30 µl aliquot of Qiagen Repli-G Midi kit buffer DLB amended with 3.5 µl of 50% Tween-20 and 3.5 µl dithiothreitol (DTT). The neutralization solution used was the “Stop” solution included with the Qiagen Repli-G Midi kit. Recovery solution consisted of a 1 ml aliquot of 20 mM Tris-HCL PH 8.0/1 mM EDTA amended with 2 µl 10% Tween-20. Reaction solution consisted of 29 µl of 1x Qiagen Repli-G Midi Buffer amended with 10 µl 20 mg/ml BSA, 10 µl 25% glycerol, 1.5 µl of Eva Green dsDNA stain, and 0.5 µl 0.75 M DTT solution. 2 µl of DNA polymerase was added to reaction mixture after UV treatment.

### **08/05/2014, 11/21/2014 (Protocol 2)**:

The sample sorted was a concentrated cell suspension from the 03/10/2011 cruise, taken from 250 m depth. The lysis solution consisted of a 30 µl aliquot of Qiagen Repli-G Midi kit buffer DLB amended with 3 µl non-UV-treated DTT, added following UV treatment of the DLB solution but prior to injection of the DLB solution into the microflidics device. The neutralization solution used was the “Stop” solution included with the Qiagen Repli-G Midi kit. Reaction solution consisted of 29 µl of 1x Qiagen Repli-G Single-Cell Buffer amended with 10 µl 20 mg/ml BSA, 10 µl 25% glycerol and 2 µl Eva Green dsDNA stain. 2 µl of DNA polymerase was added to reaction mixture after UV treatment. Recovery solution consisted of a 1 ml aliquot of 10 mM Tris-HCL PH 8.0/1 mM EDTA amended with 2 µl 10% Tween-20.

### **09/18/2015 (Protocol 3)**:

The sample sorted was a concentrated cell suspension from the 07/02/2015 cruise, taken from 20 m depth. The lysis solution consisted of 0.4 N cold KOH with 100 mM DTT added following UV treatment. The neutralization solution consisted of 400 mM HCL added into a solution of 400 mM Tris-HCL (PH 8.0 before addition). Reaction solution consisted of Epicentre RepliPhi buffer, 500 µM phosphorthioated random hexamer, 5% glycerol, 5 mM DTT, 2 mg/ml BSA, 1x Eva Green dsDNA stain, and 20 U/µl Epicentre RepliPhi Phi29 phage polymerase. DTT added to lysis solution, Eva Green Stain and Phi29 Polymerase were exempted from UV treatment. Recovery solution consisted of a 1 ml aliquot of 10 mM Tris-HCL PH 8.0/1 mM EDTA amended with 2 µl 10% Tween-20.

### **11/17/2015, 12/03/2015 (Protocol 4)**:

The sample sorted was a concentrated cell suspension from collected from 20 m depth on 07/02/2015 cruise. The lysis solution consisted of 0.5 N cold KOH with 100 mM added following UV treatment. The neutralization solution consisted of 400 mM HCL added into a solution of 400 mM Tris-HCL (PH 8.0 before addition). Reaction solution consisted of 50 mM Tris-HCL PH 8.0, 50 mM KCL, 5 mM NH_4_SO_4_, 10 mM MgCl_2_, 500 µM phosphorthioated random hexamer, 5% glycerol, 20 mM DTT, 0.5 mg/ml BSA, 150 mM trehalose, 0.01% NP-40, 1x Eva Green dsDNA stain, and 20 U/µl Epicentre RepliPhi Phi29 phage polymerase. DTT added to lysis solution, Eva Green dsDNA stain, and Phi29 polymerase were exempted from UV treatment. Recovery solution consisted of a 1 ml aliquot of 10 mM Tris-HCL PH 8.0/1 mM EDTA amended with 2 µl 10% Tween-20.

### Evaluation of methods.

The estimated coverage of genome assemblies from the reaction mixture we repot in the methods was in comparison to the reactions that used the Qiagen Repli-G reagent set. Completion estimates were not able to be quantified for 5 of these products and the average completion was 21% for an additional three reactions. Using the Repli-G reagents, two assemblies had low estimated levels of completion and the average completion estimate was 40% for the other assemblies. However, a number of the older reactions using Repli-G reagents were sequenced with an average of 2.5x higher raw sequencing coverage than any of the reactions using the most recent chemistry. It is possible that the coverage biases inherent in MDA reactions might greatly affect the outcome of the final assemblies. Without the highly sequenced assemblies our average estimated coverage is 33%.

The success of single cell genome amplifications with the in-house reaction formula, which can be seen in the three successful sort dates of wild-type marine cells that took place within a relatively short period of time. This is in contrast to amplifications using the Repli-G reagents, which proved difficult to optimize for microfluidics due to their unknown composition. The consistency of the known reaction mixture is a remarkable success, despite possible shortfalls in coverage, as it allows for continuing optimization of emerging microfluidic technology.

## QPCR SCREENING

### **07/30/2013, 08/06/2013:**

QPCR was performed using an ABI 7300 machine with 27FB and 338RPL primers (27F 5'-AGA GTT TGA TCM TGG CTC AG-3'; 338RPL: 5'-GCW GCC WCC CGT AGG WGT-3') (Carlson et al., 2004). A concentration of 300 nM of each primer was used. Cycling conditions were as follows: 1 cycle of 2:00 @ 50 °C, 5:00 @ 95 °C; 60 cycles of 0:10 @ 94 °C, 0:30 @ 59 °C followed by a disassociation stage from 60 °C to 95 °C. 2 sets of 8 standards were used with concentrations spanning the range of 1x10^1 – 1x10^8 cp/µl. A detection threshold of 1000 cp/µl in the template was used for positive reactions.

### 08/05/2014, 11/21/2014:

QPCR was performed using a Roche 480 LightCycler machine with M13F- and M13R-ligated 515F and 806R primers (M13F-515F: 5'-TGT AAA ACG ACG GCC AGT GTG CCA GCM GCC GCG GTA A-3'; M13R-806R: 5'-CAG GAA ACA GCT ATG ACC GGA CTA CHV GGG TWT CTA AT-3') (Caporaso et al., 2010). A concentration of 300 nM of each primer was used. Cycling conditions were as follows: 1 cycle of 2:00 @ 50 °C, 5:00 @ 95 °C; 50 cycles of 0:15 @ 95 °C, 0:30 @ 58 °C, 0:45 @ 72 °C; 1 cycle of 5:00 @ 72 °C; followed by a disassociation stage from 60 °C to 95 °C. 3 sets of 8 standards were used with concentrations spanning the range of 1x10^2 – 1x10^9 cp/µl. A detection threshold of 1000 cp/µl in the template was used for positive reactions.

### **09/18/2015, 1/17/2015**, **12/03/2015:**

QPCR was performed using an ABI 7500 Fast machine with M13F- and M13R-ligated 515F and 806RB primers (M13F-515F: 5'-TGT AAA ACG GCC AGT GTG CCA GCM GCC GCG GTA A-3'; M13R-806RB: 5'-CAG GAA ACA GCT ATG ACC GGA CTA CNV GGG TWT CTA AT-3') (Apprill et al., 2015). A concentration of 333 nM of each primer was used. Cycling conditions were as follows: 1 cycle of 1:00 @ 50 °C, 2:00 @ 95 °C; 50 cycles of 0:15 @ 95 °C, 0:30 @ 58 °C, 0:45 @ 72 °C; 1 cycle of 5:00 @ 72 °C; followed by a disassociation stage from 60 °C to 95 °C. 3 sets of 6 standards were used with concentrations spanning the range of 1x10^2 – 1x10^7 cp/µl. A detection threshold of 100 cp/µl in the template was used for positive reactions.
