# Supplementary material for: Optofluidic Single-Cell Genome Amplification of Sub-micron Bacteria in the Ocean Subsurface
Source: Front Microbiol. 2018 Jun 8;9:1152. doi: 10.3389/fmicb.2018.01152 (PMC6003095; doi:10.3389/fmicb.2018.01152)
Supplement: Data Sheet 3 — DBSCAN implementation for potential contaminant detection in R. This document describes the semi-supervised method to examine single-amplified genome assemblies for possible contaminants. Includes an R-scripted version of protocol. [file Data_Sheet_3.ZIP › S3/S3_Document.pdf]

Supplementary material for *Optofluidic single-cell genome amplification of E01-9C-26 and other sub-micron bacteria specialized to niches in the ocean subsurface:* DBSCAN implementation for potential contaminant detection in R

Zachary Landry

December 1, 2017

Traditionally, contamination detection in single-amplified genomes (SAG) is performed through a somewhat subjective visual inspection of sequence fragments plotted along the most significant principal components (PCs) derived from a principal component analysis (PCA) of tetranucleotide frequencies (Woyke et al. 2009). There are other robust methods of excluding possible contamination, but these often rely on comparative samples (eg. the 'jackknifing' approach used in Dodsworth et al. 2013). User inspection of the PCA plot is used to identify contigs or contig fragments displaying a significantly different tetranucleotide profile from the other sequences, under the assumption that the majority of the assembled contigs represent the genome of the desired organism. To save time and manpower, a novel partially-automated method for identifying potential outliers was developed that achieved these goals and was able to consistently identify contigs that would have attracted suspicion under human inspection. An scripted version of this process is included in the supplement.

Dimensionality Reduction

Prior to implementing our analysis, sequences from all SAG contigs 2,000 bp or longer were divided into 2,000 bp fragments using a step size of 1,000 bp. Tetranucleotide frequencies of each contig were counted for each of 136 unique reverse-complemented tetramers. Tables of these statistics were previously saved for import into R.

```
> infile<-("./E01.OSU.005_k4/E01.OSU.005.txt")
> data<-read.table(infile, header=TRUE, row.names=1)
```

Metadata included in these tables is stripped from oligonucleotide counts and stored for later use.

```
> not.mer<-c("frag","scaffold","start","end")
> kmer.data<-data[,! names(data) %in% not.mer]
> not.mer.data<-data[,names(data) %in% not.mer]
```

Principal component analysis can be run using the prcomp() function included in R.

```
> pca<-prcomp(kmer.data)
```

In this application, PCA returns 136 unique PCs, ranked in order of explained variance. Frequently, PCA analyses are (somewhat arbitrarily) limited to the first 2 or three principal components, but this runs the risk of excluding possibly useful data. As you can see from the following graph of the standard deviations for each PC, the first few principal components explain much more variability than the rest. After the first few PCs, most subsequent principal components begin to explain more or less the same average amount of variance:

```
> plot(pca$sdev, col='green')
```

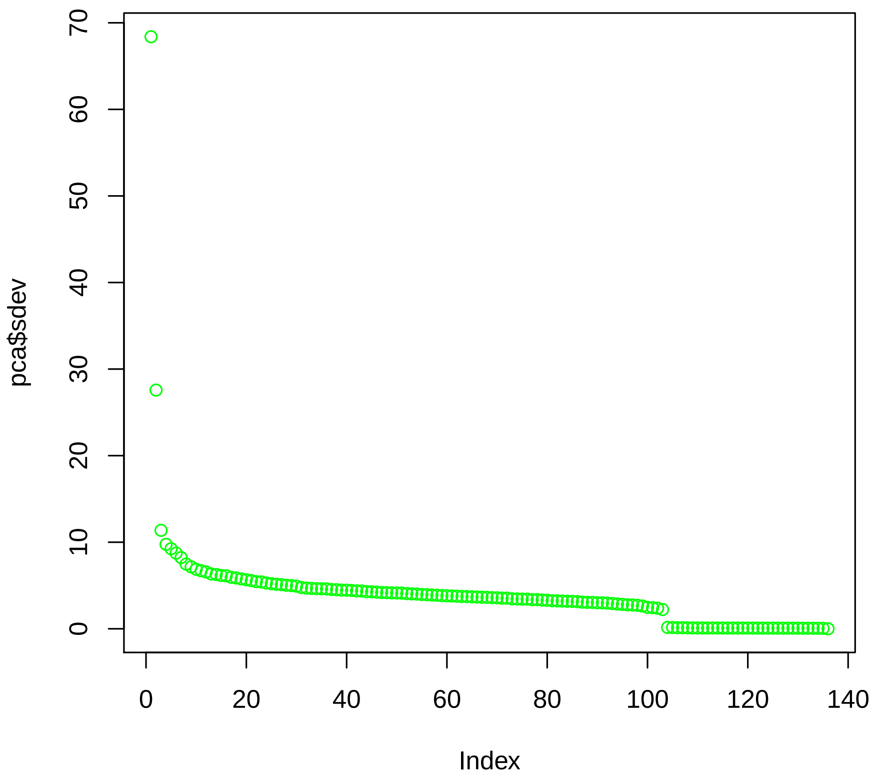

To identify the inflection point where the examination of extra principal components begin imparting only an average amount of additional understanding, and to define a restricted lower-dimensional set of informative PCs, the following approach was used. Upon

examining the PCA results of each SAG, the the difference between the standard deviations of each principal component and its previous principal component was normalized to the difference in standard deviations between the first two principal components:

```
> test<-vapply(
+   2:length(pca$sdev),
+   function(x, sdev){
+     (sdev[x-1]-sdev[x])/(sdev[1]-sdev[2])
+   },
+   0,
+   sdev=pca$sdev
+ )
```

The log-normalized version of this vector shows a more-or-less normal distribution:

```
> test<-log(test)
> hist(test, breaks=seq(0,-15,-0.5))
```

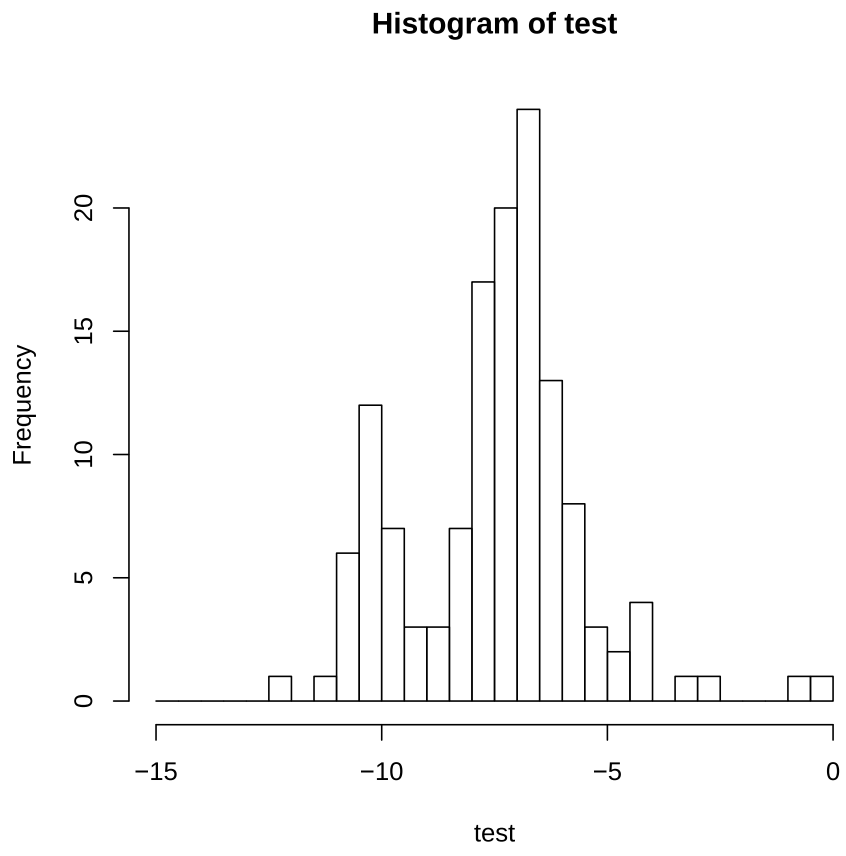

The log-normalized vector of these differences was used to compute an average difference for all principal components, where this value represented the average loss in the explanation of variance for each principal component in comparison to the previous PC. A threshold for principal components whose difference exceeds the average was defined as the mean of all differences plus the standard deviation of all differences.

```
> limit<-mean(test)+1*sd(test)
> hist(test, breaks=seq(0,-15,-0.5))
> abline(v=limit, col='red',lty=2)
```

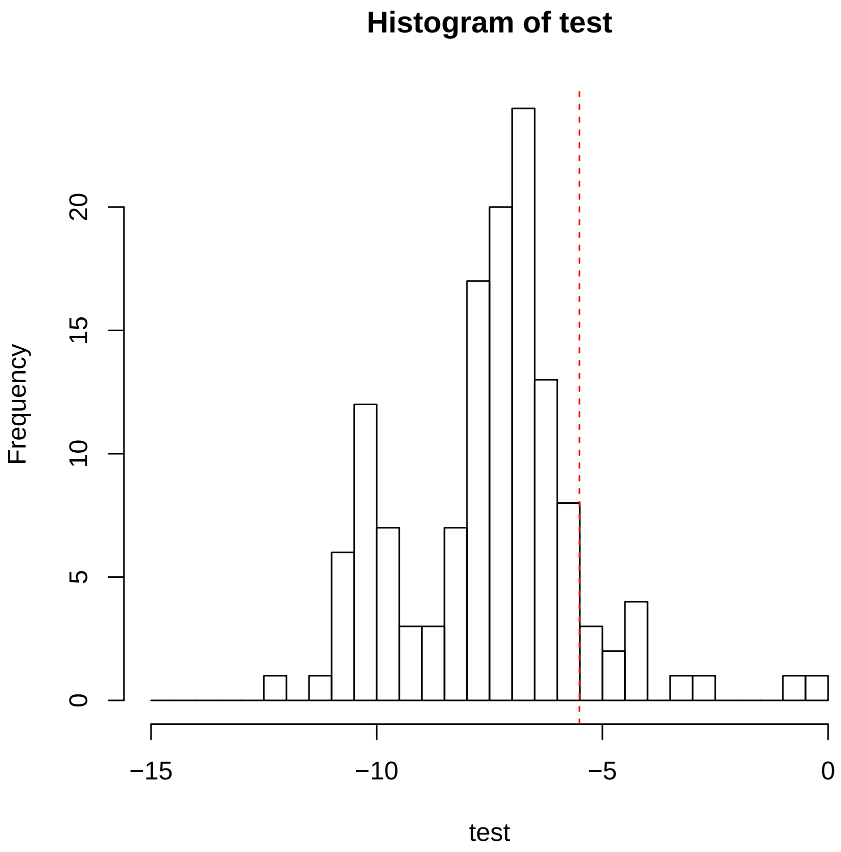

The first *n* dimensions (PCs) exceeding this threshold are reserved for further analyses, and the remainder are discarded (the first PC is always reserved by default).

```
> pcs<-c(1)
> for (i in 1:length(test)){
+         if (test[i] < limit){
+                 break
+         }
+         pcs<-append(pcs, i+1)
+ }
> keep<-pca$x[,pcs]
>
```

Below is the previously shown plot of standard deviations for each PC with the only the reserved PCs marked in green:

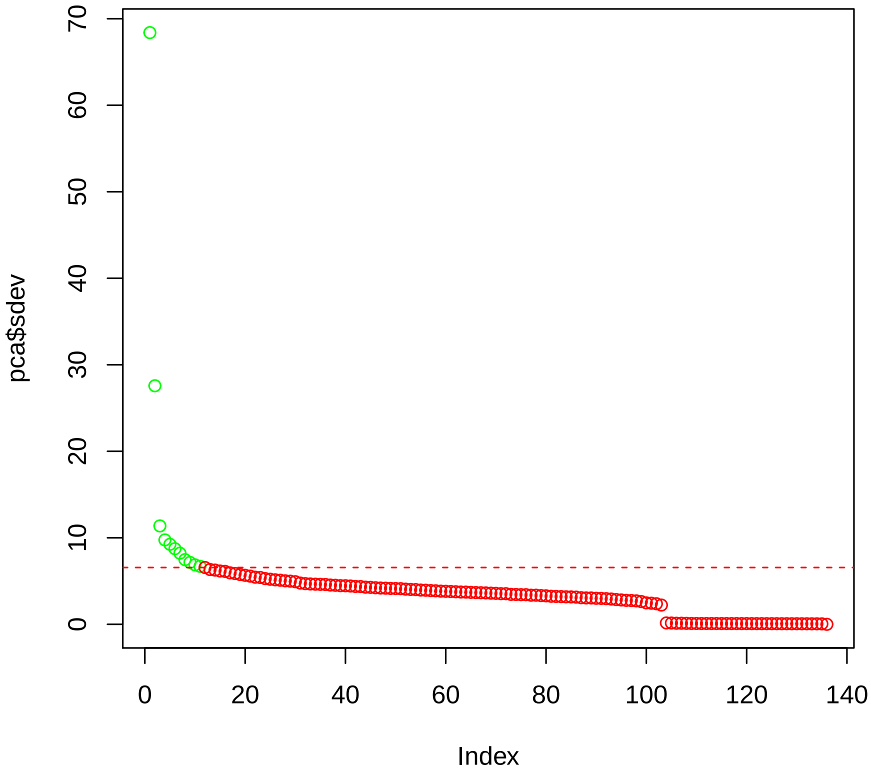

DBSCAN Implementation

The implementation of the DBSCAN clustering algorithm provided by the 'fpc' package in R was used to cluster sequence fragments and define a common profile for the majority of assembled contigs. It also serves to identify any potentially contaminated contigs. The DBSCAN algorithm was chosen over other clustering methods for three intrinsic major advantages:

- It does not require the user to provide a number of desired clusters.
- It allows for the classification of data points as outliers not belonging to any cluster.
- It is able to identify clusters of arbitrary shape or size (most important).

In lieu of a desired number of clusters, DBSCAN requires the input of two parameters:

- A minimum number of points to cluster.
- A'neighborhood' parameter defining the maximum distance between two points or clusters to be joined.

For more information concerning the inner workings of the algorithm or its implementation, please refer to Ester, et al, 1996, "A Density-Based Clustering Algorithm for Discovering Clusters in Large Spatial Datasets with Noise" or the 'fpc' documentation (https://cran.r-project.org/web/packages/fpc/fpc.pdf).

The minimum number of points in this case was defined as the minimum number of vertices needed to construct a space-filling model in n-dimensional space or $n+1$, where n is the number of principal components reserved from our dimensionality reduction described above.. The 'neighborhood' parameter was defined here as the average of the distances from point p to its $n+1$ nearest-neighbors.

```
> #recalculate distance after removing less valuable PCs
> k.dist<-dist(keep)

> #Minimum points equals the total number of kept dimensions
> #plus one, in an effort to use all spatial dimensions.
> minpts<-length(pcs)+1

> #meandist equals the mean of <minpts> nearest neighbors, for
> #every point in the dataset
> meandist<-apply(
+   as.matrix(k.dist),
+   1, function(x, n){
+     mean(sort(x[x!=0])[1:n])
+   },
+   n=minpts
+ )

> #eps (neighborhood distance) equals the average of the average
> #distances to <minpts> nearest neighbors in the dataset
> eps<-mean(meandist)
```

The effect of this is that fragments with at least $n+1$ nearest neighbors within average distance of themselves in n-dimensional space cluster together, almost always forming a large 'core' cluster containing the majority of the sequence fragments. Fragments whose nearest neighbors are further removed are marked as outliers or classified into separate clusters. Due to the properties of the DBSCAN algorithm, output clusters can be any shape or size. To execute dbscan():

```
> require(fpc)
> dbc<-dbscan(k.dist, eps, minpts, method='dist' )
```

Color-coded three-dimensional plots of the output clusters within the first three principal components are able to be produced using the 'rgl' package R and can visually inspected to confirm that no blatant outlier sequences are remaining unidentified.

```
> #colors assigned by cluster using heatmap function
> cols<-heat(dbc$cluster)
```

```
> require(rgl)
```

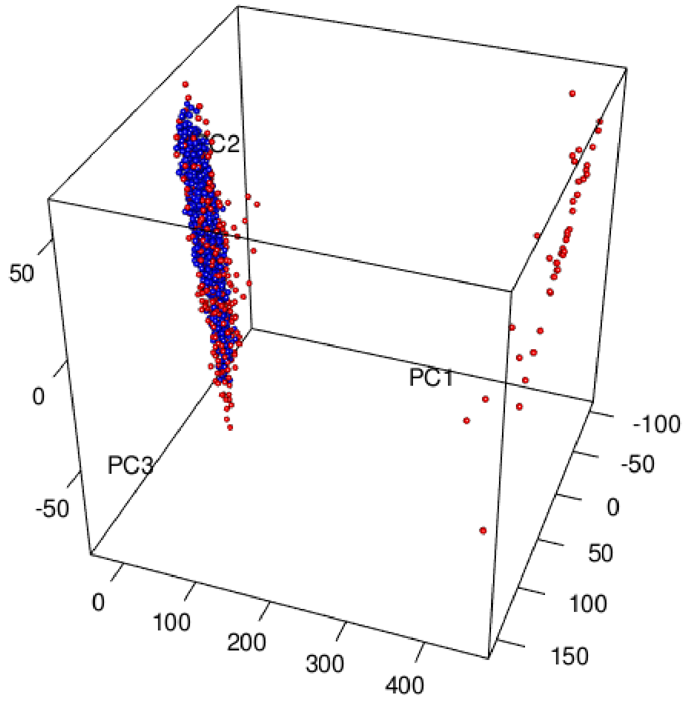

The plot above shows fragments from the 'core' cluster being shown in blue and outlier fragments shown in red. Two distinct groups of fragments can be seen here, with one group consisting mostly of 'core' cluster fragments, and the other consisting entirely of outlier fragments. This second cluster is far removed from the 'core' cluster, representing a potential contaminant. Assignment of contigs as belonging to the 'core' cluster, a secondary cluster, or as an outlier is performed based on a 2/3 consensus of sequence fragments for any given contig. Contigs for which only 1/2 - 2/3 of sequence fragments belong to a specific group are grouped with outliers. Contigs for which the assignment is to the core cluster can be marked as 'valid'.

Post-Processing and Validation

Following cluster assignments, BLASTP and BLASTN results of all protein and nucleic acid coding sequences are used to validate some possibly misclassified "potential contaminant" sequences as well as sequences less than 2,000 bp in length. Coding sequences for these contigs are assigned a taxon based on their top hit to the NCBI RefSeq NR and RefSeq Genome databases. Contigs for which the majority of the taxa coincide with taxa found on multiple contigs in the 'valid' contig set can be reassigned. This step is important,

as due to the fragmentation of SAG assemblies, many informative genes are found on contigs less than 2,000 bp, and structural RNAs such as 16S rRNA or tRNAS found alone on a single contig can be misclassified due to diverged oligonucleotide profile. Using our example here, a modified version of the three-dimensional PCA plot can be seen below showing fragments from 'valid' *contigs* (as opposed to individual fragment assignments) in blue and fragments from unclassified *contigs* in red:

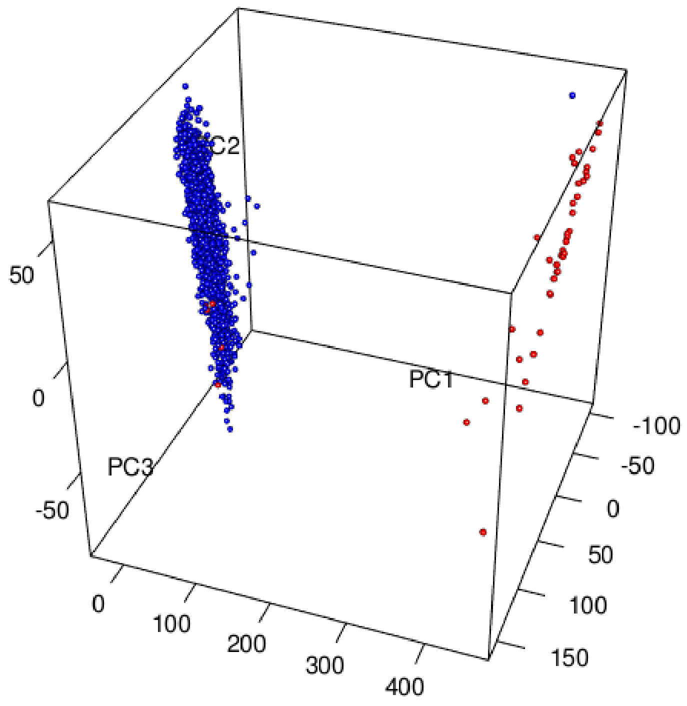

Following reclassification, any remaining contigs not marked as 'valid' will be removed from future analyses. Additionally taxa assignments of 'valid' contigs can be checked to ensure that they fit the expected taxonomic range of the organism, as previously identified by 16S phylogeny.
