# Supplementary material for: Optofluidic Single-Cell Genome Amplification of Sub-micron Bacteria in the Ocean Subsurface
Source: Front Microbiol. 2018 Jun 8;9:1152. doi: 10.3389/fmicb.2018.01152 (PMC6003095; doi:10.3389/fmicb.2018.01152)
Supplement: Data Sheet 4 — Relative abundance of E01-9C-26 Gammaproteobacterial 16S genes from the TARA Oceans dataset. Ribosomal rRNA genes from this group are ubiquitously present in mesopelagic samples throughout the dataset, suggesting that the clade is numerically relevant to this environment throughout the world's oceans. [file Data_Sheet_4.pdf]

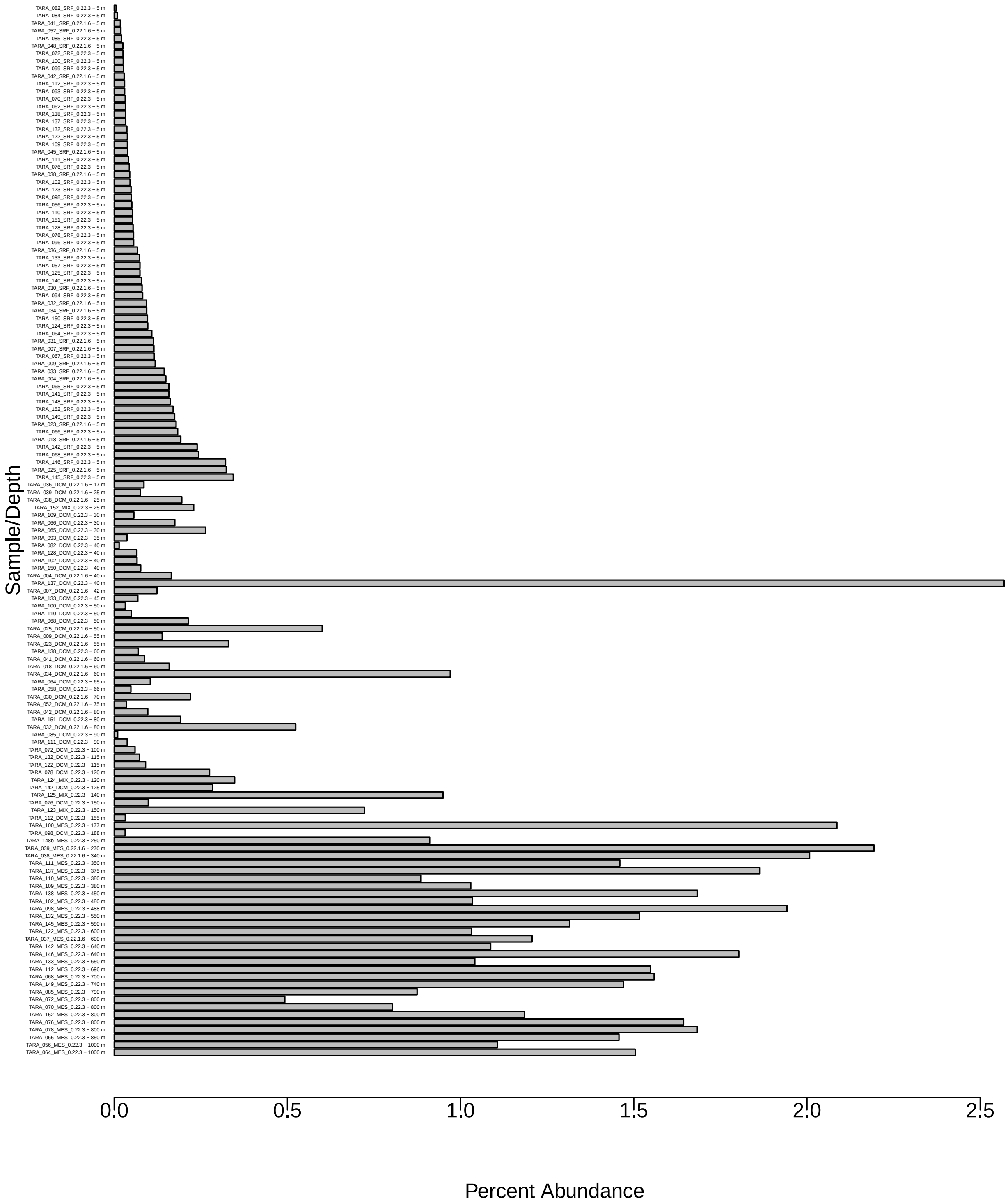

Figure S1. Relative abundance of E01-9C-26 Gammaproteobacterial 16S genes from the TARA Oceans dataset. Ribosomal rRNA genes from this group are ubiquitously present in mesopelagic samples throughout the dataset, suggesting that the clade is numerically relevant to this environment throughout the world's oceans.
